# Hospital Festivals and Meetings

**Published:** 1895-03-23

**Authors:** 


### HOSPITAL FESTIVALS AND MEETINGS.

### ROYAL HOSPITAL FOR DISEASES OF THE CHEST, CITY ROAD.

In consequence of his continued illness the Lord Mayor was unable to preside at the annual general meeting which was held on Wednesday, March 13th, and his place was taken by Mr. Sheriff Hand.

The report, which was taken as read, announced that certain sanitary alterations having become imperative early in the year, the matter had been put into the hands of Mr. Rogers Field, the eminent sanitary engineer, and the work had been thoroughly carried out. The alterations had necessitated the closing of the wards for four and a half months, and of the out-patient department for two months, during which time, by the kindness and courtesy of the Victoria Park and other hospitals, the inconvenience to the patients had been much lessened. The total cost of the work would amount to about £2,900. Notwithstanding the issue of two special appeals, the total expenditure had exceeded the income by nearly £1,500, and in order to meet the cost of the drainage works £1,800 had been borrowed of the bankers.

Dr. Gilbart Smith supported the movement in favour of the adoption of the report. He thought an annnal report, dealing only with bare facts, left out much of the real life of a hospital. For instance, this report did not mention the hours and hours spent by the chairman, Mr. De la Rue, and Lord Charles Bruce over the affairs of the hospital, the time devoted by the secretary in looking after every detail of every department, the devotion shown by the nursing staff, the matron and her nurses, and the medical staff. Neither did it show the inner life in the wards, the patients snatched from death or awaiting the last summons, the joy when it was possible to send a man or woman home, able once more to earn bread for the family. With regard to the facts mentioned in the report, the closing of the hospital for four and a half months had been a great grief to all interested in it, but it was now opened again under very much better auspices. The out-patient department was greatly improved, and the hospital generally in a better position than ever before for the work it was doing.

Colonel Makins spoke as to the sanitary improvements, and Mr. De la Rue and the Rev. J. H. Rose both bore cordial testimony, from their personal knowledge among the patients, of the great kindness invariably shown by the medical staff.

After a few words from Mr. Sheriff Hand, who said he had had the greatest pleasure in being present, and in going through the wards, the meeting closed with the usual votes of thanks.

The rooms and corridors were gay with flowers and palms, and the matron, and her nurses in their pretty red uniforms, dispensed refreshing cups of tea to the visitors as they left the board room.

The council announce that a concert will be given by Mr. Moberley's string orchestra at St. James's Hall on May 17th, at half-past eight p.m., of which the entire proceeds will go to the hospital, and they hope governors will keep that evening vacant.

### THE DENTAL HOSPITAL OF LONDON.

The 37th annual meeting of this institution was held at the hospital on Wednesday, March 13th, Mr. Henry Harben, J.P., a vice-president, in the chair. The committee of management congratulated the governors on the continued usefulness and prosperity of the charity, but regretted the fact that the majority of the donations still came from the members of the dental profession instead of from the public. That the hospital continued to afford great benefit to the suffering poor was shown by 58,499 cases having been treated during the year 1894, being 3,174 in excess of those of the previous year, and 39,240 in excess of the number treated in 1874, in which year the hospital was moved from Soho Square to its present site. It had been found absolutely necessary, in consequence of the increased number of patients, the crowded state of the waiting-rooms, the absence of passage room, and the insanitary, inadequate, and badly-fitted condition of the present building, additional premises being unattainable, to build a new hospital, and a special appeal was issued for the necessary funds, and the committee had the pleasure to acknowledge £11,223 16s. 4d. The value of the present site was estimated at £15,000 to £20,000 (but this sum could not be realised until the new building was erected), making together about £26,000, and as it had been estimated that the cost of the site and the new hospital would be about £40,000, there remained a balance of £14,000 to be collected, which sum the committee asked the public to speedily contribute. The new site was purchased, and the houses upon it let at such rentals as paid the interest on the money advanced by the bank. Dentistry was one of the few specialties of the healing art that was justified in possessing a special hospital, the Committee of Management therefore had no hesitation in making very urgent appeals—firstly, for the funds necessary to erect the new hospital, and, secondly, for an increased number of annual subscribers to enable the charity to continue its usefulness to the poor, and to meet the cost of maintenance, the hospital being absolutely free from any endowment, and relying entirely upon the free-will offerings of the charitable public to be enabled to carry on its useful work.

### ROYAL HOSPITAL FOR CHILDREN AND WOMEN, WATERLOO BRIDGE ROAD.

The annual meeting of the friends and supporters of this hospital was held at the Mansion House on March 15th. The Lord Mayor being still unable to attend public functions, the chaplain of the hospital, the Rev. A. Powell, took the chair. The Lady Mayoress was present, but as so frequently has been the case at such meetings this season, the general attendance was very small. Letters of regret were received from the Archbishop of Canterbury, the Bishop of Rochester, and others. In the report, read by the secretary, the committee regretted that the financial depression had necessitated selling of stock to the amount of £750 for current expenses, leaving still a debt of about £260. An urgent appeal to the public was therefore imperative. The work of the hospital had been fully maintained during the past year, and there had been a great increase in the surgical work. The Nurses' Home in Stamford Street continued to be successful, the private nursing staff giving every satisfaction. The committee earnestly appealed for help. The chairman moved the adoption of the report, and pointed out the immense need of such an institution in South London, the largest continuous area of poverty in the known world. There were 51 beds in the hospital, which were nearly always full, and nearly 8,000 out-patients were treated yearly, figures meaning an amount of relief from suffering hardly to be realised. It was to be remembered that the number of residents in this district able to help the funds grew smaller every year, as people who could afford it were more prone to live out of London. The ordinary income was below the necessary expenditure by some £2,000 a year. Mr. Powell, and other speakers who followed, spoke warmly of their own intimate personal knowledge of the trust reposed in the hospital by the poor people for whose relief it existed, and of their hearty appreciation of its work. The Treasurer referred in grateful terms to the valued support of the City companies. Before the meeting ended, subscriptions and donations to the amount of about £120 was announced, and a motion was passed to the effect that the hospital was in every possible way worthy of increased support.

### THE COUNTY HOSPITAL, YORK.

The annual court of the governors of this hospital was held in the board-room at noon on Tuesday, the 12th inst. Amongst those present were the Very Rev. the Dean of York, the Right Hon. the Lord Mayor of York, Major Allenby, Mr. Alderman Milward, Mr. C. Sellers, the manager of the York United Gas Company, and several other gentlemen. The Dean of York having been unanimously voted to the chair, the business of the day was proceeded with. The Chairman, in moving the adoption of the annual report, referred in brief terms to the chief events of the year. He pointed out that the hospital had suffered financially with most of the other charitable institutions, owing to the depression in trade, and that, unfortunately, a diminished income had to be announced. But, on the other hand, he was pleased to say that, without impairing the efficacy of the work of the hospital, a great reduction in the expenditure had been effected. He then went on to say that the Committee, through the generosity of a friend and supporter of the hospital, had been enabled to lay out very prettily as a promenade for the convalescent patients a piece of waste land adjoining the hospital. This work they had long felt was desirable, but had, owing to want of means, been unable to do. In conclusion, he referred to the following changes in the staff of the hospital: Early in the year the Lady Superintendent (Miss Forrest), owing to private business, had to send in her resignation; Miss Gwyn, late of the Bath Hospital, Harrogate, and the Worcester Infirmary, being appointed in her place. The Secretary and Manager (Mr. C. E. Pinfold) and Dr. Coles (Senior House Surgeon) also resigned; being succeeded by Mr. F. W. Howell, of the "Dreadnought" Seamen's Hospital, Greenwich, and Mr. C. Hodgson, the Junior House Surgeon, respectively; Mr. R. N. Luce, of Guy's Hospital filling the junior post. The proceedings terminated with a cordial vote of thanks to the chairman, moved by the Lord Mayor, and seconded by Mr. Alderman Milward.

